# Photoinhibition of Photosystem I Provides Oxidative Protection During Imbalanced Photosynthetic Electron Transport in *Arabidopsis thaliana*

**DOI:** 10.3389/fpls.2019.00916

**Published:** 2019-07-12

**Authors:** Yugo Lima-Melo, Vicente T. C. B. Alencar, Ana K. M. Lobo, Rachel H. V. Sousa, Mikko Tikkanen, Eva-Mari Aro, Joaquim A. G. Silveira, Peter J. Gollan

**Affiliations:** ^1^Department of Biochemistry and Molecular Biology, Federal University of Ceará, Fortaleza, Brazil; ^2^Molecular Plant Biology, Department of Biochemistry, University of Turku, Turku, Finland

**Keywords:** photosystem I, photosynthesis, ROS, CO_2_ fixation, photoinhibition, P700, redox

## Abstract

Photosynthesis involves the conversion of sunlight energy into stored chemical energy, which is achieved through electron transport along a series of redox reactions. Excess photosynthetic electron transport might be dangerous due to the risk of molecular oxygen reduction, generating reactive oxygen species (ROS) over-accumulation. Avoiding excess ROS production requires the rate of electron transport to be coordinated with the capacity of electron acceptors in the chloroplast stroma. Imbalance between the donor and acceptor sides of photosystem I (PSI) can lead to inactivation, which is called PSI photoinhibition. We used a light-inducible PSI photoinhibition system in *Arabidopsis thaliana* to resolve the time dynamics of inhibition and to investigate its impact on ROS production and turnover. The oxidation state of the PSI reaction center and rates of CO_2_ fixation both indicated strong and rapid PSI photoinhibition upon donor side/acceptor side imbalance, while the rate of inhibition eased during prolonged imbalance. PSI photoinhibition was not associated with any major changes in ROS accumulation or antioxidant activity; however, a lower level of lipid oxidation correlated with lower abundance of chloroplast lipoxygenase in PSI-inhibited leaves. The results of this study suggest that rapid activation of PSI photoinhibition under severe photosynthetic imbalance protects the chloroplast from over-reduction and excess ROS formation.

## Introduction

Light is vital for photosynthesis, but when supplied in excess it can damage the photosynthetic apparatus and cause photo-oxidative stress. This condition occurs during states of photosynthetic imbalance, when the electron pressure in the photosynthetic electron transport chain exceeds the capacity of reducing power consumption by sink pathways, which is usually associated with stressful environmental conditions. As a result, transient or sustained production of reactive oxygen species (ROS) can occur. Excessive accumulation of ROS can impair metabolic homeostasis through oxidative damage to cells because of their high reactivity with lipids, proteins, and nucleic acids (McCord, 2000; Apel and Hirt, 2004; Munns, 2005; Sharma et al., 2012). On the other hand, ROS play an important role in signaling pathways essential for acclimation to environmental conditions (for recent reviews, see Mittler, 2017; Czarnocka and Karpiński, 2018; Mullineaux et al., 2018). ROS can induce signaling responses directly, or indirectly by driving redox changes that modulate signaling networks (de Souza et al., 2017; Exposito-Rodriguez et al., 2017; Noctor et al., 2018; Souza et al., 2018). In addition, oxidation by-products, including oxidized lipids and pigments, transduce signals (Mueller et al., 2008; Mosblech et al., 2009; López et al., 2011; Ramel et al., 2012; Satoh et al., 2014). Because ROS are harmful at high concentrations, but at the same time are important for signaling and plant acclimation, the precise control of ROS concentrations is critical for metabolic homeostasis. Accordingly, plants control photosynthetic ROS production by regulating light-harvesting and electron transport (reviewed in Tikkanen and Aro, 2014), in particular through protonation of the thylakoid lumen that requires the proton gradient regulation 5 (PGR5) protein (Munekage et al., 2002; Suorsa et al., 2012). In the chloroplast stroma, ROS concentrations are regulated by antioxidant systems involving numerous redox enzymes, including the superoxide dismutases (SOD), which catalyze the dismutation of superoxide radical (O${}_{2}^{•-}$) to hydrogen peroxide (H_2_O_2_), that in turn can be subsequently reduced to water by ascorbate peroxidases (APX) and other peroxidases through the Foyer–Halliwell–Asada cycle, using ascorbate (ASC), glutathione (GSH) and thioredoxins as electron donors (Asada, 1999; Foyer and Shigeoka, 2011).

A central consequence of photo-oxidative stress is inactivation of the photosystems, a phenomenon known as “photoinhibition” (reviewed in Aro et al., 1993; Gururani et al., 2015). Photoinhibition decreases photosynthetic capacity and can therefore be deleterious to plant growth and yield (Takahashi and Murata, 2008; Kato et al., 2012; Simkin et al., 2017). Photosystem I (PSI) is particularly resistant to photoinhibition under oxidative stress conditions, due to the high efficacy of protective mechanisms that regulate the flow of electrons to the PSI donor side, including non-photochemical quenching (NPQ), lumen pH-dependent regulation of cytochrome *b6f* activity, and even PSII photoinhibition (reviewed in Tikkanen and Aro, 2014). Electron consumption at the PSI acceptor side through the Calvin-Benson cycle, photorespiration, cyclic and pseudo-cyclic electron flow are also protective factors that prevent PSI over-reduction (Yamori, 2016; Li et al., 2018).

Despite this, PSI photoinhibition occurs under specific conditions of excessive electron pressure from PSI electron donors on the lumenal side, or/and insufficient capacity of electron acceptors at the stromal side. Under these stress conditions, reduction of O_2_ produces O${}_{2}^{•-}$ that can inactivate PSI iron-sulfur (FeS) clusters and cause PSI inhibition (Sonoike and Terashima, 1994; Sonoike, 1995; Takagi et al., 2016; Tiwari et al., 2016). In contrast to PSII, the recovery of inhibited PSI has been shown to occur very slowly, over several days (Barth et al., 2001; Kudoh and Sonoike, 2002; Huang et al., 2010; Lima-Melo et al., 2019). PSI photoinhibition in wild type plants has been observed under low irradiance at chilling temperatures, due to down-regulation of stromal electron sinks (Inoue et al., 1986; Terashima et al., 1994; Tjus et al., 1998; Zhang and Scheller, 2004) as well as under fluctuating light (Kono et al., 2014). On the other hand, resistance against PSI photoinhibition can be induced by acclimation to low temperature and high light conditions (Ivanov et al., 1998, 2012). Severe PSI photoinhibition can occur when pH-dependent control of electron transport is inactivated, such as in plants lacking the PGR5 protein (Munekage et al., 2002; Nandha et al., 2007; Suorsa et al., 2012; Tiwari et al., 2016). High light treatment of the *pgr5* mutant of *Arabidopsis thaliana* has provided an inducible model for PSI inhibition that has been used to study the mechanisms of PSI damage and the impacts of PSI photoinhibition on photosynthesis and metabolism of plants (Tiwari et al., 2016; Gollan et al., 2017; Lima-Melo et al., 2019). Exposure of *pgr5* to sudden increases in light intensity causes PSI FeS cluster damage (Tiwari et al., 2016) and degradation of PSI subunit proteins (Suorsa et al., 2012; Lima-Melo et al., 2019).

Although several studies have shown that PSI photoinhibition is triggered by ROS (Sonoike and Terashima, 1994; Sonoike, 1995; Sejima et al., 2014; Takagi et al., 2016), the correlation between PSI photoinhibition and ROS metabolism is not clear. In the current study, we investigated the dynamics of PSI photoinhibition in *pgr5* mutants under high light stress, and the relationship between PSI photoinhibition and ROS accumulation associated with occurrence of oxidative stress at the whole leaf level. Our data suggest that PSI photoinhibition is a mechanism to prevent excessive ROS production in order to minimize oxidative stress, at the expense of carbon assimilation and normal growth.

## Materials and Methods

### Plants, Growth and Treatment Conditions

The *proton gradient regulation 5* (*pgr5*) mutant plants of *A. thaliana* L. Heynh. ecotype Columbia, which are in the *glabrous 1* genetic background (Munekage et al., 2002), were used alongside wild-type (WT) *glabrous 1* plants in all experiments. Plants were grown for 6 weeks in a growth chamber at 23°C, relative humidity 60%, 8/16 h of light/dark photoperiod under constant white light of 125 μmol photons m^–2^ s^–1^ (GL). For high light (HL) treatments, plants were shifted from GL to 1,000 μmol photons m^–2^ s^–1^ for 1 h, while control groups were kept under GL. Experiments were repeated at least twice and at least three independent replicates were used in every experiment.

### Photochemical and Gas Exchange Measurements

Photosystem II and photosystem I photochemistry were measured simultaneously using a Dual-PAM-100 system (Walz, Germany) based on chlorophyll *a* fluorescence (Schreiber et al., 1995) and P700 absorbance (Klughammer and Schreiber, 1998). Detached leaves were analyzed after 30 min of dark acclimation. Gas exchange measurements (net CO_2_ assimilation, *A*; transpiration, *E*; stomatal conductance, *g*_*S*_; and internal CO_2_ concentration, *C*i) were performed in detached leaves after 15 min dark acclimation, using a LI-6400XT Portable Infrared Gas Analyzer (IRGA) equipped with an LED source (LI-COR Biosciences, United States). The environmental conditions inside the IRGA chamber were: 400 ppm CO_2_, 1.0 ± 0.2 kPa VPD and 25°C. For the net CO_2_ assimilation (*A*) time-course assay, *A* was recorded every 15 s during changes of light intensity between GL and HL with the following protocol: 15 min of dark, 30 min of GL, 60 min of HL, 60 min of GL, 30 min of HL. For rapid light curves, a PPFD gradient of five increasing steps (0, 50, 125, 500, and 1,000 μmol photons m^–2^ s^–1^) was used. Gas exchange data were logged after IRGA parameters reached steady-state values after the start of each light intensity (usually around 120 s). The water use efficiency (WUE) and the maximum carboxylation efficiency were calculated as *A*/*E* and *A*/*C*i, respectively.

### Leaf Membrane Damage and H_2_O_2_ Content

Leaf membrane damage (MD) was estimated through the electrolyte leakage method (Blum and Ebercon, 1981). Detached leaves (5 plants, 2 leaves from each plant) were placed in tubes containing deionized water and incubated in a shaking water bath at 25°C for 24 h. After measuring electric conductivity (L1), the solution was heated at 95°C for 1 h and then cooled to 25°C, after which the second electric conductivity (L2) was measured. Membrane damage was calculated as MD = (L1/L2) × 100. The H_2_O_2_ content was quantified using the Amplex Red Hydrogen Peroxide/Peroxidase Assay Kit (Life Technologies, Carlsbad, CA, United States) according to the manufacturer protocols. Fresh leaves were ground to a fine powder in liquid N_2_ followed by the addition of potassium phosphate buffer (final concentration of 100 mM; pH 7.5). The absorbance at 560 nm was measured to quantify the H_2_O_2_ concentration (Zhou et al., 1997) and results were expressed as μmol H_2_O_2_ g^–1^ fresh weight (FW).

### Histochemical Detection of Superoxide and Hydrogen Peroxide

Nitroblue tetrazolium (NBT) and diaminobenzidine (DAB) staining were performed for *in situ* detection of superoxide (O${}_{2}^{•-}$) and hydrogen peroxide (H_2_O_2_) accumulation, respectively, in leaves, as previously described (Ogawa et al., 1997; Thordal-Christensen et al., 1997). High light-treated leaves were detached and submerged in tubes containing DAB solution [4.67 mM DAB; 1% isopropanol (v/v) and 0.1% Triton (v/v)] or NBT solution [0.1% NBT (m/v) and 10 mM NaN_3_ in 10 mM potassium phosphate buffer, pH 7.8], both protected from light, and incubated for 24 h. For NBT staining, leaves were moved to petri dishes containing water and treated with light (approximately 20 μmol m^–2^ s^–1^) for 30 min prior to the end of the 24 h incubation. DAB- and NBT-stained leaves were then incubated in a bleaching solution [TCA 0.15% (m/v) diluted in ethanol:chloroform (4:1 v/v)] for 48 h. Stained leaves were then submerged in 80% ethanol and heated (70°C) in a water bath for 15 min, followed by several washes with 80% ethanol until complete removal of pigments. Leaves were then dried and photographed.

### Lipid Peroxidation (TBARS Content and Autoluminescence Imaging)

Lipid peroxidation was estimated according to the formation of thiobarbituric acid-reactive substances (TBARS; Heath and Packer, 1968). Fresh leaves were ground to a fine powder in liquid N_2_ followed by the addition of TCA [final concentration of 5% (w/v)]. After centrifugation at 12,000 × *g* for 15 min, 500 μl of the supernatants were immediately diluted in 2 ml of a solution containing 0.5% (w/v) of thiobarbituric acid (TBA) and 20% (w/v) of TCA and heated at 95°C in a water bath for 1 h. After cooling to 25°C, the solutions were centrifuged at 10,000 × *g* for 5 min and supernatants were collected for absorbance readings at 532 and 660 nm using a spectrophotometer. The absorption values at 660 nm obtained from blank samples without leaf tissue were subtracted. The concentration of TBARS was calculated using the absorption coefficient of the thiobarbituric acid-malondialdehyde complex (TBA-MDA), which is 155 mM^–1^ cm^–1^, and the results were expressed as nmol TBA-MDA g^–1^ FW. Lipid peroxidation was also assessed by the autoluminescence of leaves and rosettes according to the method described in Birtic et al. (2011). Detached leaves or rosettes treated with GL (control), HL or physical wounding with forceps were incubated in darkness for 2 h before the luminescence signal was collected over 20 min on an electrically cooled charged-couple device (CCD) camera, using an IVIS Lumina II system (Caliper Life Sciences, United States).

### Protein Extraction and Enzymatic Activity Assays

Fresh leaves were ground to a fine powder in liquid N_2_ followed by the addition of potassium phosphate buffer (final concentration of 100 mM; pH 7.0) containing EDTA (final concentration of 1 mM). The homogenate was centrifuged at 15,000 × *g* at 4°C for 15 min, and the resulting supernatant was used for determination of all enzymatic activities. Total soluble protein content was measured according to Bradford (1976), and all the activities were expressed on the basis of protein. All enzymatic activities were determined spectrophotometrically. Superoxide dismutase (SOD; EC 1.15.1.1) activity was determined based on inhibition of nitro blue tetrazolium chloride (NBT) photoreduction (Giannopolitis and Reis, 1977). The reaction mixture contained 75 μM NBT, 20 μM riboflavin, and 100 μl of the protein extract, all diluted in 50 mM potassium phosphate buffer (pH 6.0) containing 1 mM EDTA in a final volume of 2 ml, which was incubated under illumination (30 μmol photons m^–2^ s^–1^) at 25°C for 5 min. The absorbance was measured at 540 nm. One SOD activity unit (U) was defined as the amount of enzyme required to inhibit 50% of the NBT photoreduction, expressed as U mg^–1^ protein min^–1^. Catalase (CAT; EC 1.11.1.6) activity was based on the reduction of H_2_O_2_ (Beers and Sizer, 1952; Havir and McHale, 1987). The reaction mixture contained 20 mM H_2_O_2_, and 25 μl of the protein extract, all diluted in 50 mM potassium phosphate buffer (pH 7.0) in a final volume of 1.5 ml. The reaction was started by adding the protein extract and the decrease in absorbance at 240 nm at 30°C was monitored for 300 s. CAT activity was calculated using the molar extinction coefficient of H_2_O_2_ (40 mM^–1^ cm^–1^) and expressed as μmol H_2_O_2_ mg^–1^ protein min^–1^. APX (EC 1.11.1.11) activity was measured based on the oxidation of ascorbate (ASC) (Nakano and Asada, 1981) in a reaction mixture containing 0.45 mM ASC, 3 mM H_2_O_2_, and 50 μl of the protein extract, all diluted in 100 mM potassium phosphate buffer (pH 7.0) containing 1 mM EDTA in a final volume of 1.5 ml. The reaction was started by adding the H_2_O_2_ solution and the decrease in absorbance at 290 nm at 25°C was monitored for 300 s. APX activity was expressed as μmol ASC mg^–1^ protein min^–1^. Monodehydroascorbate reductase (MDHAR; EC 1.6.5.4) activity was assayed based on the generation of monodehydroascorbate (MDHA) free radicals by ascorbate oxidase (AO; 1.10.3.3) and following oxidation of NADH (Hossain et al., 1984) in a reaction mixture containing 0.1 mM NADH, 2.5 ASC, 0.84 units/ml AO, and 75 μl of the protein extract. The reaction mixture was adjusted to 725 μl with 50 mM Tris-HCl buffer (pH 7.6). The reaction was started by adding the AO solution and the decrease in absorbance at 340 nm at 25°C was monitored for 300 s. MDHAR activity was calculated using the extinction coefficient of NADH (6.2 mM^–1^ cm^–1^) and expressed as nmol NADH mg^–1^ protein min^–1^. Dehydroascorbate reductase (DHAR; EC 1.8.5.1) activity was assayed based on the oxidation of GSH (Nakano and Asada, 1981) in a reaction mixture containing 2.5 mM GSH, 0.2 mM dehydroascorbate (DHA), and 50 μl of the protein extract, all diluted in 50 mM potassium phosphate (pH 7.0) in a final volume of 1.5 ml. The reaction was started by adding the DHA solution and the increase in absorbance at 265 nm at 25°C was monitored for 300 s. DHAR activity was expressed as nmol NADH mg^–1^ protein min^–1^.

### Gene Expression Analysis

Plants were treated with GL and HL, after which leaves were detached and frozen in liquid N_2_. Leaf samples contained four leaves from individual plants. Frozen leaves were ground to a powder in liquid N_2_ and total RNA was purified using TRIsure (Bioline, United States), according to the protocol supplied, with an additional final purification in 2.5 M LiCl overnight at −20°C. RNAseq libraries were constructed, and libraries were sequenced in 50 bp single-end reads using Illumina Hiseq 2500 technology (BGI Tech Solutions, Hong Kong). Reads were aligned to the reference genome (*A. thaliana* TAIR 10) using Strand NGS 2.7 software (Agilent, United States). Aligned reads were normalized and quantified using the DESeq R package. Gene expression fold changes were calculated using a two-way ANOVA test on triplicate samples (*n* = 3) with Benjamini–Hochberg *p*-value correction to determine the false discovery rate (FDR) for each gene.

### Western Blotting

Leaf tissue was ground to a powder in liquid nitrogen and then incubated in 20 mM Tris buffer (pH 7.8) containing 2% SDS for 20 min at 37°C, followed by 5 min centrifugation at 15,000 × *g*. The supernatant containing total leaf protein was used for Western blotting. 10 μg of total protein were separated on SDS-PAGE gels containing 12% acrylamide, transferred to polyvinylidene difluoride (PVDF) membranes and blotted with polyclonal antibodies against LOX-C antiserum (AS07 258; Agrisera).

## Results

### High Light Rapidly Induces PSI Photoinhibition in *pgr5* Mutants

**FIGURE 1:**
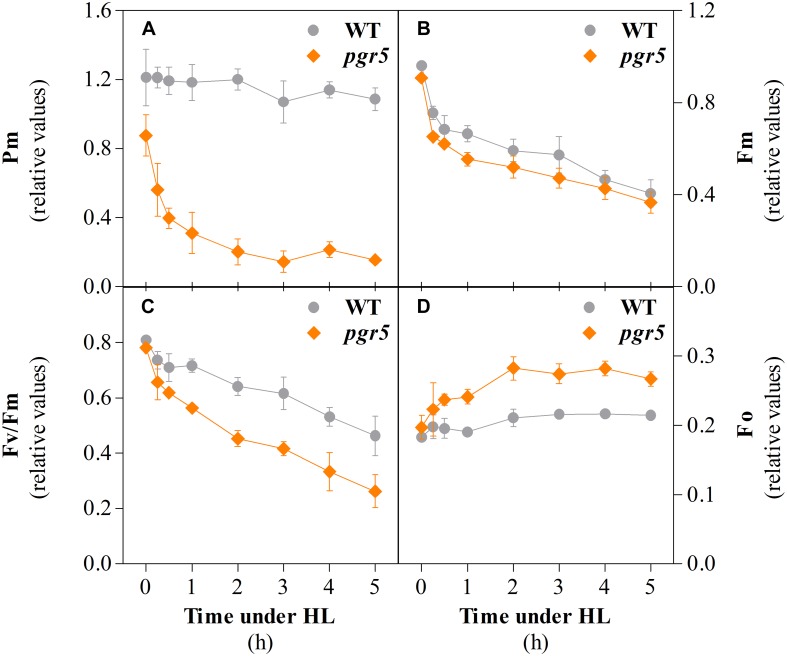
Parameters associated with PSI and PSII integrity in wild type (WT) and *pgr5* mutants during treatment with high light (HL). Maximum oxidizable P700 (*P*_*m*_, **A**); maximum chlorophyll *a* fluorescence (*F*_*m*_, **B**); maximum efficiency of PSII (*F*_*v*_/*F*_*m*_, **C**); minimum chlorophyll *a* fluorescence (*F*_*o*_, **D**) measured in detached leaves of WT and *pgr5* plants grown under a photosynthetic photon flux density of 125 μmol m^–2^ s^–1^ and treated with 1,000 μmol m^–2^ s^–1^ for 5 h. Error bars show standard deviation among replicates (*n* = 4). Significant differences between genotypes are indicated by non-overlapping error bars (Student’s *t*-test, *p* < 0.05).

Photosynthetic parameters were monitored in leaves of wild-type (WT) and *pgr5* mutant plants that were grown under 125 μmol photons m^–2^ s^–1^ (growth light; GL) and then exposed to 1,000 μmol photons m^–2^ s^–1^ (high light; HL). Chlorophyll *a* fluorescence and P700 absorbance were measured during 5 h HL treatments. The PSI photoinhibition levels were estimated through the evaluation of the maximum oxidation of P700 at the PSI reaction center (*P*_*m*_). Before the HL treatment, the average *P*_*m*_ value of WT leaves (1.2) was almost 30% higher than that of *pgr5* leaves (0.85; see 0 h in Figure 1A). The *P*_*m*_ value of WT leaves remained virtually unchanged through the 5 h HL treatment, whereas the same parameter in *pgr5* decreased to 60% of the pre-treatment level after only 15 min under HL, with further decreases to 45 and 35% after 30 min and 1 h HL, respectively. *P*_*m*_ in HL-treated *pgr5* reached a steady-state value of around 0.15 (20% of pre-treatment *P*_*m*_) after 3 h of HL treatment.

PSII photoinhibition was evaluated by monitoring the decrease of the maximum chlorophyll *a* fluorescence (*F*_*m*_) during the same time-course experiment. The *F*_*m*_ values before the onset of the HL treatment were almost identical in WT and *pgr5* (Figure 1B). After 15 min, *F*_*m*_ values decreased to 0.75 and 0.65 in WT and *pgr5*, respectively, and then showed a steady decline over the course of the 5 h HL treatment in both genotypes. In contrast, the HL-induced decline in the calculated *F*_*v*_/*F*_*m*_ (maximum quantum efficiency of PSII) parameter was substantially greater in the *pgr5* mutant (Figure 1C), which corresponded to significantly higher levels of minimum chlorophyll *a* fluorescence (*F*_*o*_) after 30 min HL exposure, when compared to WT (Figure 1D).

### Time-Resolved Diminution of CO_2_ Assimilation During PSI Photoinhibition

**FIGURE 2:**
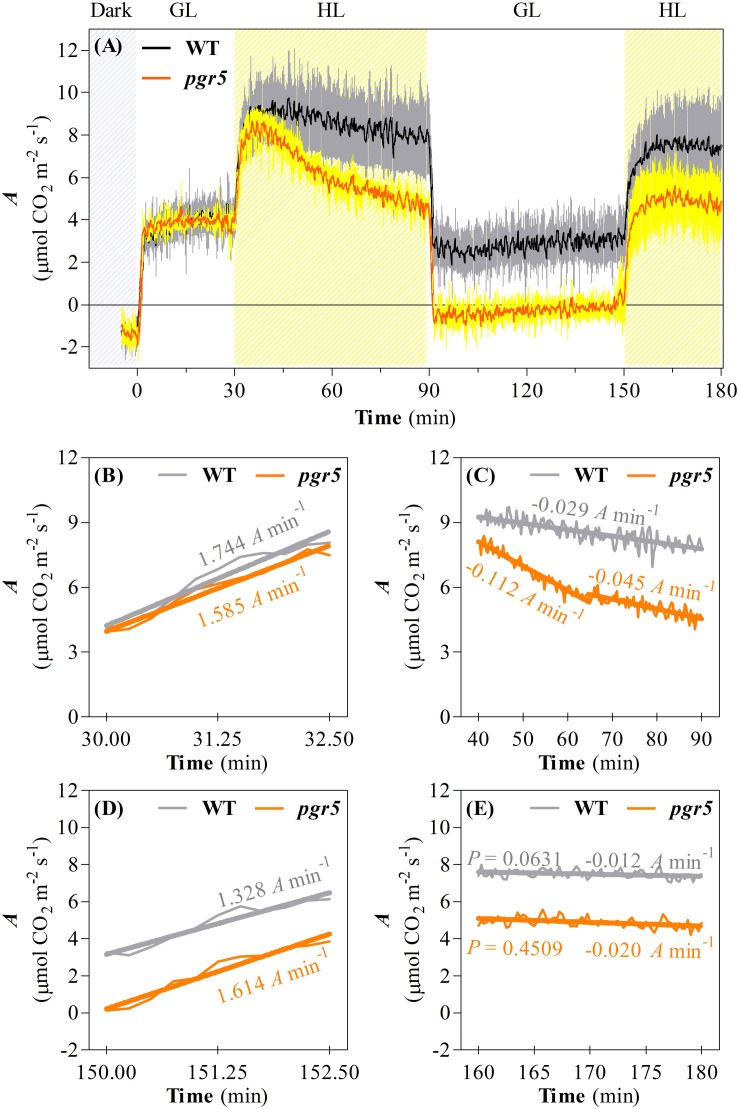
Changes in net CO_2_ assimilation during a time-course PSI photoinhibition. **(A)** CO_2_ assimilation (*A*) in leaves of WT and *pgr5* mutants during changes of light intensity between growth light (GL; 125 μmol m^–2^ s^–1^) and high light (HL; 1,000 μmol m^–2^ s^–1^). Hatched gray and hatched yellow sections indicate dark and HL treatments, respectively. Error margins are shown in gray (WT) and yellow (*pgr5*) and indicate standard deviation among replicates (*n* = 6). Significant differences between treatments and genotypes are indicated by non-overlapping error bars (Student’s *t*-test, *p* < 0.05). Linear regression lines of changes in *A* drawn in key steps of the time-course like the onset of the first HL treatment **(B)**, during the first HL treatment **(C)**, the onset of the second HL treatment **(D)**, and during the second HL treatment **(E)**. *P*-values were calculated to estimate if the slopes are significantly different from zero. All *P*-values were lower than 0.0001, except where shown in **(E)**.

In order to investigate the consequences of progressive HL-induced photoinhibition of PSI on net CO_2_ assimilation rate (*A*) and respiration in WT and *pgr5* mutant plants, these processes were evaluated during cycles of GL and HL exposure (Figure 2). In both genotypes, similar rates of CO_2_ assimilation and day-time respiration (measured by CO_2_ evolution in the dark) were observed in GL-treated plants (Figure 2A). During the first minutes of the transition from GL to HL, *A* increased at a rate of approximately 1.6–1.7 μmol CO_2_ m^–2^ s^–1^ *per* min in both WT and *pgr5* plants (Figure 2B). After approximately 10 min in HL, WT *A* decreased until the end of the first hour of HL treatment at a rate of approximately 0.03 μmol CO_2_ m^–2^ s^–1^ *per* min, while *A* decline in *pgr5* during the HL treatment was far more rapid than in the WT, especially during the early phase of HL exposure (0.11 μmol CO_2_ m^–2^ s^–1^ *per* min) compared to the latter phase (0.05 μmol CO_2_ m^–2^ s^–1^ *per* min) of treatment (Figure 2C). At the end of the 1 h HL treatment, *A* was 40% lower in *pgr5* mutants than in WT (Figures 2A,C). Under a second phase of GL following the HL treatment, *A* in WT leaves was slightly lower than the level observed in the first GL phase prior to the HL treatment, while the rate in HL-treated *pgr5* mutants was approximately 0 μmol CO_2_ m^–2^ s^–1^ (Figure 2A). A second HL treatment after 1 h GL induced another rapid increase in CO_2_ fixation for both genotypes, and in each case the maximum initial rates under the second treatment were approximately equivalent to the rates observed before the end of the previous HL treatment (7.5 μmol CO_2_ m^–2^ s^–1^ in WT and 5 μmol CO_2_ m^–2^ s^–1^ in *pgr5*), corresponding to approximately 35% lower CO_2_ fixation in *pgr5* than in WT during the second HL treatment (Figure 2A). Notably, the rate of increase in *A* during the second HL treatment was slower in both WT and *pgr5* (0.13 and 0.16 μmol CO_2_ m^–2^ s^–1^ *per* min, respectively; Figure 2D) in comparison to the rates of increase during the first HL treatment (1.74 and 1.58 μmol CO_2_ m^–2^ s^–1^ *per* min, respectively; Figure 2B). The rate of decline in *A* during the second HL treatment was similar between WT and *pgr5* (Figure 2E), and smaller than that observed during the first HL treatment (Figure 2C).

**FIGURE 3:**
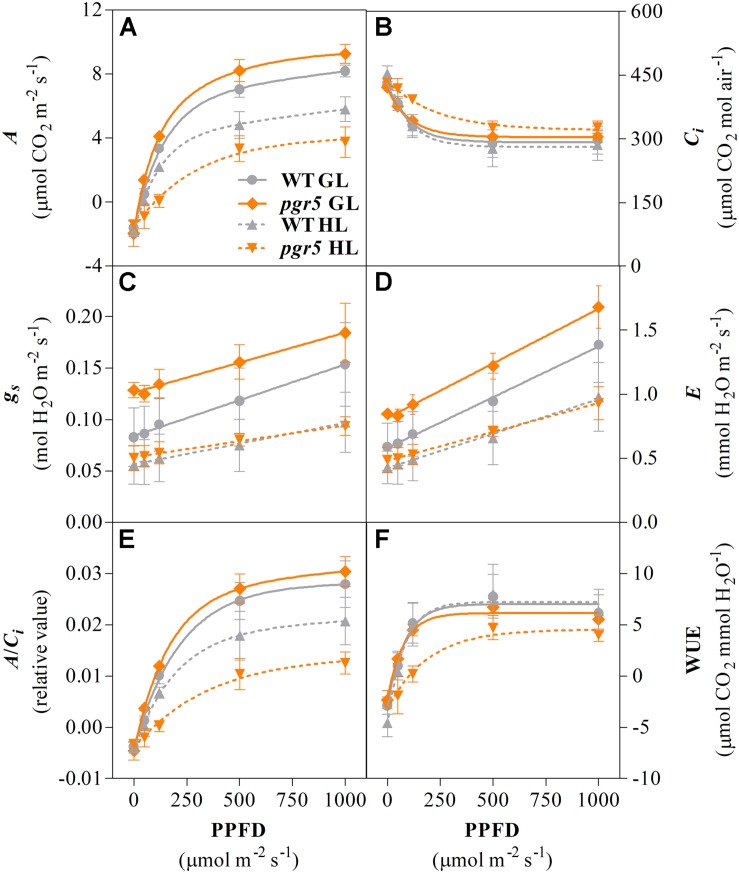
Gas exchange response to increasing light intensity. Net CO_2_ assimilation rate (*A*, **A**); internal CO_2_ concentration (*C*_*i*_, **B**); stomatal conductance rate (*g*_*s*_, **C**); transpiration rate (*E*, **D**); maximum carboxylation efficiency (*A*/*C*_*i*_, **E**); water use efficiency (WUE, **F**) measured in leaves of WT and *pgr5* plants previously treated with growth light (GL; 125 μmol m^–2^ s^–1^) or high light (HL; 1,000 μmol m^–2^ s^–1^) for 1 h. Error bars show standard deviation among replicates (*n* = 4). Significant differences between treatments and genotypes are indicated by non-overlapping error bars (Student’s *t*-test, *p* < 0.05).

Analyses of gas exchange in WT and *pgr5* plants pre-treated with GL or HL for 1 h were conducted using light-response curves to investigate the effects of PSI photoinhibition under different irradiances. CO_2_ assimilation rates over increasing light intensities were similar in both WT and *pgr5* plants treated with GL and were substantially decreased in both genotypes after 1 h HL treatment (Figure 3A). Significantly lower *A* values were observed in HL-treated *pgr5* compared to HL-treated WT, especially in the region of the curve measured under irradiances below 200 μmol photons m^–2^ s^–1^ (Figure 3A). Higher internal CO_2_ concentration (*C*_*i*_) was recorded in HL-treated *pgr5* at the lowest irradiances of the light curve when compared to all other plants, while there were no significant differences in *Ci* at high irradiances (Figure 3B). Stomatal conductance (*g*_*s*_) values were higher in GL-treated *pgr5* when compared to GL-treated WT, and were substantially lower in both genotypes after the HL treatment, compared to GL-treated plants (Figure 3C). No differences in *g*_*s*_ values between the genotypes were observed after the HL treatment. The changes in transpiration rate (*E*) over the light curve were similar to that observed for *g*_*s*_ (Figure 3D). The trends observed in the maximum carboxylation efficiency (*A*/*C*_*i*_)-PPFD curve were similar to those in the *A*-PPFD curve (Figures 3A,E, respectively), although the difference between the HL-treated *pgr5* and the other groups was more evident, as a consequence of the higher *C*_*i*_ values under low irradiances (Figure 3B). Water use efficiency (WUE) was strikingly lower in the HL-treated *pgr5* mutants when measured under low irradiances, compared to the other treatments (Figure 3F), reflecting the very low *A* measured in those leaves (Figure 3A).

### ROS Accumulation, and Activities and Expression of Antioxidant Systems After PSI Photoinhibition

**FIGURE 4:**
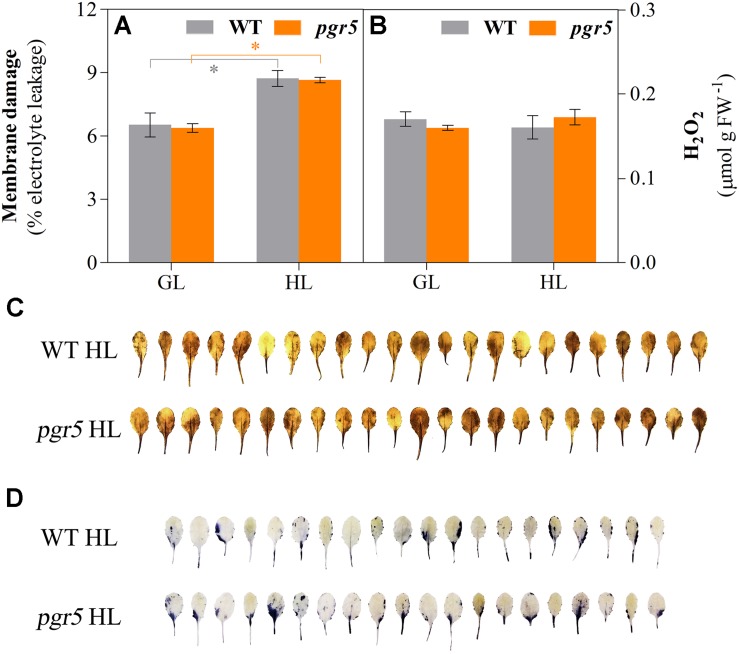
Indicators of oxidative stress in WT and *pgr5* mutants under growth light or after high light treatment. Membrane damage **(A)** and H_2_O_2_ content **(B)** in leaves of WT and *pgr5* mutants treated with growth light (GL; 125 μmol m^–2^ s^–1^) or high light (HL; 1,000 μmol m^–2^ s^–1^) for 1 h. Error bars show standard error among replicates (*n* = 4). Significant differences between treatments and genotypes are indicated by asterisks (Student’s *t*-test, *p* < 0.05). 3,3-diaminobenzidine (DAB, **C**) and nitro-blue tetrazolium (NBT, **D**) staining to assess the accumulation of H_2_O_2_ and superoxide accumulation, respectively, in detached leaves of HL-treated WT and *pgr5* plants.

To explore the relationship between PSI photoinhibition and accumulation of ROS, several markers of oxidative stress were evaluated in HL-treated leaves of WT and *pgr5* mutants. Membrane damage, estimated through electrolyte leakage, increased significantly after 1 h of HL treatment in both genotypes, compared to the GL controls; however, no difference was detected between WT and *pgr5* mutants in either condition (Figure 4A). Spectrophotometric measurements of H_2_O_2_ concentrations in leaf tissue showed no difference between GL and 1 h HL treatments or between genotypes (Figure 4B). Qualitative *in situ* assessments of H_2_O_2_ and superoxide (O${}_{2}^{•-}$) accumulation in HL-treated leaves by 3,3′-diaminobenzidine (DAB) and nitro-blue tetrazolium (NBT) staining, respectively, also showed no obvious differences between WT and *pgr5* in terms of accumulation of these ROS (Figures 4C,D).

**FIGURE 5:**
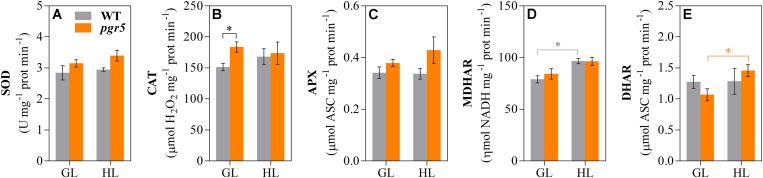
Activities of Foyer–Halliwell–Asada cycle enzymes. Activities of superoxide dismutase (SOD, **A**); catalase (CAT, **B**); ascorbate peroxidase (APX, **C**); monodehydroascorbate reductase (MDHAR, **D**) and dehydroascorbate reductase (DHAR, **E**) were measured in total leaf extracts of WT and *pgr5* mutants treated with growth light (GL; 125 μmol m^–2^ s^–1^) or high light (HL; 1,000 μmol m^–2^ s^–1^) for 1 h. Activities are normalized to protein content. Error bars show standard error among replicates (*n* = 4). Significant differences between treatments and genotypes are indicated by asterisks (Student’s *t*-test, *p* < 0.05).

**FIGURE 6:**
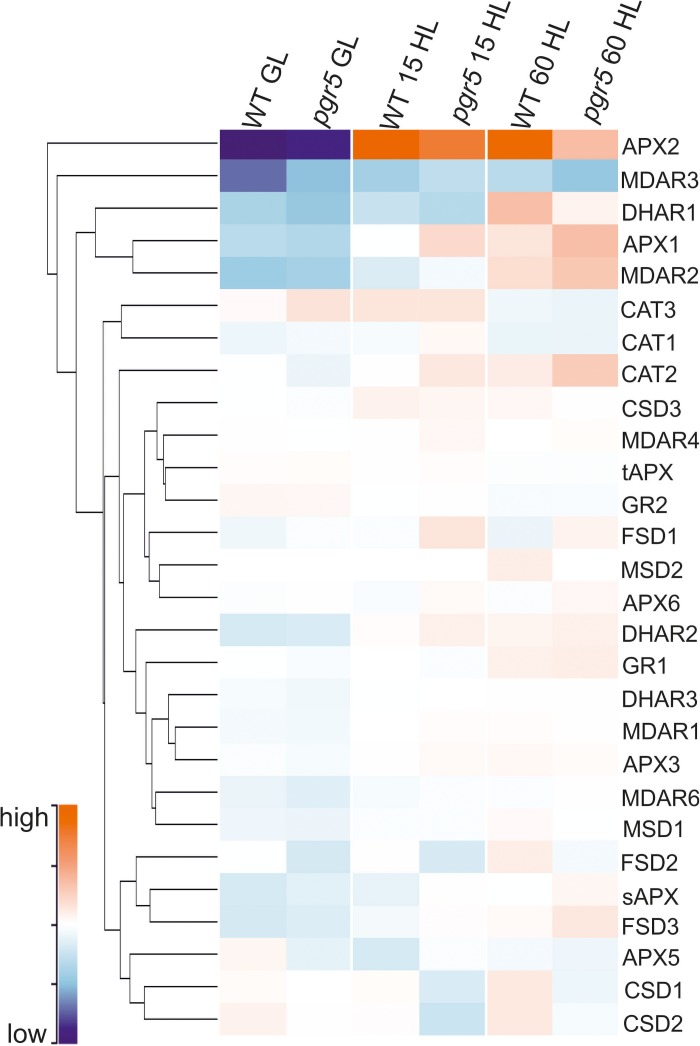
Clustered heatmap showing normalized abundance of transcripts encoding enzymes in the Foyer–Halliwell–Asada cycle. Samples show gene expression in WT and *pgr5* mutant plants under growth light (GL; 125 μmol m^–2^ s^–1^) and after 15 and 60 min in high light (HL; 1,000 μmol m^–2^ s^–1^). Color intensities indicate transcript abundance, according to the key. APX, ascorbate peroxidase; MDAR, monodehydroascorbate reductase; DHAR, dehydroascorbate reductase; CAT, catalase; CSD, Cu/Zn-superoxide dismutase; tAPX, thylakoidal ascorbate peroxidase; GR, glutathione reductase; FSD, Fe-superoxide dismutase; MSD, Mn-superoxide dismutase; sAPX, stromal ascorbate peroxidase.

Activities of SOD, CAT, APX, MDHAR, and DHAR were measured in leaves to assess any effects of PSI photoinhibition on ROS scavenging capacity. Overall, the results showed slightly higher enzyme activities in *pgr5*, in comparison to WT, in both light conditions (Figure 5). Total leaf CAT activity was significantly higher in *pgr5* than in WT under GL (Figure 5B), while total DHAR activity showed a significant increase in HL-treated *pgr5*, compared to GL-treated *pgr5*, which was not evident in WT (Figure 5E). Changes in the expression of genes involved in the Foyer–Halliwell–Asada cycle were assessed in WT and *pgr5* plants prior to HL treatment, as well as after 15 min and 1 h HL exposure. Most genes were upregulated by HL treatment in both WT and *pgr5* plants, with only minor differential expression between genotypes in most cases. Despite the similar trend of HL-induced expression in both genotypes, APX2, DHAR1 and the SOD enzymes CDS1, CDS2, and FSD2, were down-regulated in *pgr5* under HL relative to WT levels (Figure 6). Conversely, APX1, CAT2 and FSD1 were upregulated in HL-treated *pgr5* compared to the WT.

**FIGURE 7:**
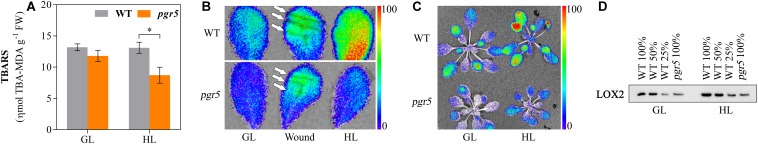
Lipid oxidation in *pgr5* mutants after high light treatment. Content of thiobarbituric acid-reactive substances (TBARS; **A**), autoluminescence imaging **(B,C)**, and Western blotting detection of chloroplast lipoxygenase (LOX2) abundance **(D)** in WT and *pgr5* mutants treated with growth light (GL; 125 μmol m^–2^ s^–1^) and high light (HL; 1,000 μmol m^–2^ s^–1^) for 1 h. Luminescence intensities **(B,C)** correspond to color scales as shown. Luminescence images overlay photographs of the same leaf/rosette samples. Wounding of leaves is indicated by white arrows in **(B)**. Error bars show standard error among replicates (*n* = 4). Significant differences between treatments and genotypes are indicated by asterisks (Student’s *t*-test, *p* < 0.05).

Assessment of thiobarbituric acid-reactive substances (TBARS) content provides an indication of lipid peroxidation. TBARS detected in GL-treated *pgr5* did not differ significantly from GL-treated WT, while after 1 h HL treatment TBARS content in *pgr5* was markedly lower than GL and WT levels (Figure 7A). Autoluminescence imaging showed that levels of lipid oxidation in leaves and rosettes increased in WT after 1 h HL treatment, in comparison to GL-treated plants, but a corresponding increase was not detected in HL-treated *pgr5* (Figures 7B,C). In contrast, strong autoluminescence signals were detected in both genotypes after mechanical wounding of leaves (Figure 7B). Western blots showed increases in the abundance of chloroplast lipoxygenase (LOX2) in both WT and *pgr5* after 1 h HL treatment. However, substantially lower LOX2 abundance was detected in both GL- and HL-treated *pgr5* leaves, in comparison to WT controls (Figure 7D), corroborating the results of TBARS tests (Figure 7A).

## Discussion

Oxidative stress in plants is closely linked to photosynthetic activity, as the transfer of photosynthetic excitation or electrons to oxygen can lead to the overproduction of ROS (for a recent review, see Mullineaux et al., 2018). Excess ROS production resulting from disturbed photosynthetic redox homeostasis is the cause of photodamage to both PSII and PSI, although the mechanisms of damage and repair differ considerably between the two photosystems and distinct intersystem regulation is evident. For example, it has become well established that PSII damage can serve as a photoprotective mechanism by preventing over-reduction and inactivation of downstream factors, especially PSI (Tikkanen et al., 2014; Huang et al., 2016). The current work suggests that rapid PSI photoinhibition under severe photosynthetic imbalance can also prevent excessive ROS production and oxidative damage.

PSI photoinhibition under disturbed redox homeostasis is associated with insufficient stromal acceptor capacity and increased utilization of O_2_ as an alternative electron acceptor, leading to formation of O${}_{2}^{•-}$ that can inactivate PSI iron-sulfur (FeS) clusters (reviewed in Sonoike, 2011). Protection from PSI photoinhibition is especially dependent on functional pH-dependent regulation of electron flow to PSI during increased irradiance (Suorsa et al., 2012; Kono et al., 2014; Tiwari et al., 2016; Gollan et al., 2017; Lima-Melo et al., 2019; Yamamoto and Shikanai, 2019). In the current work, a large decrease in *P*_*m*_ within the first minutes of exposure of *pgr5* mutants to HL shows that PSI photoinhibition occurs rapidly upon the onset of imbalance between the PSI donor and acceptor sides. This rapid inhibition suggests that the normal levels of antioxidant activity measured in *pgr5* (Figure 5) were not sufficient to mitigate ROS-induced PSI damage within the initial stages of imbalance. These results support other findings that showed that chloroplast antioxidant scavengers cannot prevent PSI photoinhibition under conditions of donor/acceptor side imbalance (Sejima et al., 2014; Zivcak et al., 2015a, 2015b; Takagi et al., 2016).

**FIGURE 8:**
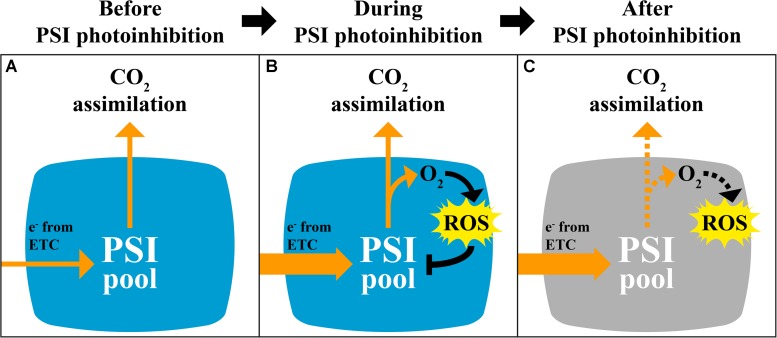
Hypothetical scheme describing the mechanism and impact of PSI photoinhibition on CO_2_ fixation and ROS production. **(A)** Under constant conditions, electron pressure at the donor and acceptor sides of PSI is balanced due to partially oxidized state of P700 and stromal electron carriers. Excess ROS production is minimal. **(B)** During the first minutes of electron imbalance between PSI donor and acceptor sides, such as during exposure of *pgr5* mutants to high light, or during low light and cold temperature (Sonoike and Terashima, 1994) or fluctuating light (Kono et al., 2014), limited stromal electron acceptor capacity leads to increased reduction of molecular oxygen. Increased ROS production leads to inactivation of PSI, presumably through oxidation of FeS clusters. **(C)** Partial inactivation of the PSI pool leads to decreased electron flow to the stroma, which limits the capacity for CO_2_ fixation, especially under low light, and presumably decreases excess ROS production to protect downstream electron acceptors.

A slower rate of *P*_*m*_ decline in *pgr5* after 30 min in HL, and the eventual stabilization of *P*_*m*_ after 2 h (Figure 1), indicated progressive decrease in ROS-induced PSI inactivation induced by decreasing intensity of stromal over-reduction. Again, this could not be attributed to any improvement in stromal ROS scavenging in *pgr5* (Figure 5) and was also not associated with decreased abundance of ROS after 1 h HL (Figure 4). Instead, a slower rate of PSI inhibition was likely directly related to alleviation of electron pressure on stromal acceptors caused by inactivation of PSI electron transport (described in Figure 8). This observation highlights the protective nature of PSI photoinhibition against over-production of ROS in the chloroplast stroma, which has been previously suggested (Tikkanen and Aro, 2014). Furthermore, in the absence of adequate pH-dependent photosynthetic control, as in the *pgr5* mutant, the extent of PSI inhibition appears to correlate to the level of imbalance between PSI donor and acceptor sides, which was high during the initial stages of HL and diminished as PSI inhibition progressed. In this way, PSI photoinhibition can be seen to support PSI donor side regulation in preventing oversupply of reductants to the stromal acceptor side. The idea that PSI photoinhibition is sensitive to the extent of photosynthetic imbalance was also evident in the changes in CO_2_ assimilation in *pgr5* during the HL treatments. A rapid rate of decline during the first 15 min of HL (Figure 2) corresponded with rapid PSI photoinhibition (Figure 1A), while slower decline during the latter part of the HL treatment correlated with a slower decrease in *P*_*m*_ during this phase of treatment. During the second HL treatment, rapid *A* decline was not observed in *pgr5* (Figure 2, 150–180 min), presumably because PSI inhibition from the previous HL exposure had effectively “pre-set” PSI activity to suit the capacity of stromal acceptors at 1,000 μmol photons m^–2^ s^–1^.

Although PSI inhibition can protect against over-production of stromal ROS during conditions of insufficient stromal acceptor capacity, PSI damage is a major impediment to carbon metabolism under normal growth conditions (Gollan et al., 2017; Lima-Melo et al., 2019). In the current study, this was especially clear in the diminished CO_2_ fixation in HL-treated *pgr5* plants under subsequent GL (Figures 2, 3A). This phenomenon was not due to decreased availability of CO_2_ in *pgr5* leaves, as internal CO_2_ concentration (*C*_*i*_) in *pgr5* plants was equivalent to WT levels, and higher in HL-treated *pgr5* plants. Instead, the light-dependent effect on *A* in PSI-inhibited plants reflects PSI quantum efficiency, where the highly reduced state of P700 under low light is exacerbated by PSI damage, while more P700^+^ is formed under HL due to more active PSI electron transport, in combination with high light-activated electron transport regulation (Baker et al., 2007; Lima-Melo et al., 2019). Despite the positive effects of HL, CO_2_ assimilation rates in PSI-inhibited plants remained lower than WT controls during the second HL treatment (Figure 2). Considering these results, we expect that the generation of O${}_{2}^{•-}$ during a subsequent HL treatment would also have been diminished in plants with inhibited PSI, although this was not specifically tested. Slightly higher stomatal conductance (*g*_*s*_) in *pgr5* mutants compared to WT under GL correlated with higher transpiration rates (*E*) in the mutant under GL, while lower water use efficiency (WUE) in HL-treated *pgr5* reflected low levels of CO_2_ fixation (Figure 3). Abnormal gas exchange in *pgr5* leaves may be related to the influence of plastoquinone (PQ) reduction state on stomatal opening and regulation of WUE in response to light (Busch, 2014; Głowacka et al., 2018), given that over-reduction of the PQ pool has been demonstrated in *pgr5* mutants, especially after HL-treatment (Nandha et al., 2007; Munekage et al., 2008; Suorsa et al., 2012; Kono et al., 2014; Gollan et al., 2017; Lima-Melo et al., 2019).

Decreased production O${}_{2}^{•-}$ and H_2_O_2_, generated from O_2_ reduction and O${}_{2}^{•-}$ dismutation, respectively, was previously observed in *pgr5* mutant seedlings exposed to fluctuating light stress (Suorsa et al., 2012). In addition, H_2_O_2_-related gene expression was negatively affected in HL-stressed *pgr5* plants (Gollan et al., 2017). These results were attributed to increased antioxidant capacity in *pgr5* (Suorsa et al., 2012) and/or decreased O_2_ reduction by inactivated PSI (Gollan et al., 2017). However, results of the current study demonstrated that ROS accumulation and oxidative stress after severe PSI photoinhibition was not substantially different from HL-stressed leaves with functional PSI, despite oxidative damage that was seen to occur in both genotypes by increases in electrolyte leakage after HL treatment (Figure 4A). Our results showing no apparent over-accumulation of H_2_O_2_ or O${}_{2}^{•-}$ in HL-treated *pgr5* leaves, compared to WT, may differ from previous results because we measured ROS levels after completion of HL treatments, rather than assaying accumulation of ROS during stress treatments (Suorsa et al., 2012). It is likely that foliar O${}_{2}^{•-}$ and H_2_O_2_ contents did increase during the first minutes under HL in both WT and *pgr5* plants, but were returned to basal levels during the 1 h treatment, as has been previously demonstrated (Galvez-Valdivieso et al., 2009; König et al., 2018). Efficient H_2_O_2_ scavenging during HL relies on the activity of the Foyer–Halliwell–Asada cycle for redox turnover of ascorbate and GSH (reviewed in Foyer and Shigeoka, 2011), which appeared to function normally in HL-treated *pgr5* plants according to similar transcript levels and activities of enzymes of the cycle (Figures 5, 6). An exception was DHAR, which showed increased total activity in HL-treated *pgr5* leaves, despite the expression of the mitochondrial DHAR1 isoform being significantly down-regulated in this condition. DHAR converts DHA to ascorbate using electrons from the reduced form of GSH, suggesting a higher accumulation of DHA in *pgr5* during HL stress. Similarly, expression of APX2 was significantly down-regulated in *pgr5* after HL treatment, as previously reported (Gollan et al., 2017). Induction of Arabidopsis APX2 expression is dependent on the occurrence of photosynthetic electron transport (Karpiński et al., 1997, 1999; Chang et al., 2004; Galvez-Valdivieso et al., 2009), which is lower in the case of severe PSI photoinhibition. Interestingly, the total activity of catalase (CAT) in *pgr5* leaves was higher under non-stress conditions than in WT leaves, correlating with increased expression of peroxisomal CAT3 in these conditions.

PSI photoinhibition in *pgr5* plants appeared to have a substantial negative effect on lipid oxidation, as measured by quantification of TBARS content and autoluminescence of leaves and rosettes. We previously detected no difference in lipid oxidation between WT and *pgr5* plants that were treated with severe high light stress that also caused chlorophyll bleaching in affected leaves of both genotypes (Gollan et al., 2017). In the current study, exposure to 1,000 μmol photons m^–2^ s^–1^ for 1 h did not bleach leaves, and this treatment revealed substantially lower levels of lipid oxidation in *pgr5* in comparison to WT. Lipid peroxides can be formed in the chloroplast either non-enzymatically, through the reaction between singlet oxygen (^1^O_2_) and unsaturated lipids (reviewed in Laloi and Havaux, 2015), or enzymatically through the activity of lipoxygenase (LOX) enzymes (reviewed in Wasternack and Hause, 2013). Non-enzymatic lipid peroxidation is associated with ^1^O_2_ formation in PSII, especially under conditions of PSII over-reduction (Triantaphylidès et al., 2008). PSI photoinhibition is known to increase excitation pressure on PSII (Suorsa et al., 2012; Kono et al., 2014; Lima-Melo et al., 2019), suggesting that higher non-enzymatic lipid oxidation may be expected in HL-treated *pgr5*. On the contrary, decreases in HL-induced lipid oxidation observed in *pgr5* likely relate to the low abundance of chloroplast-localized lipoxygenase LOX2, which was evident in both GL- and HL-treated plants (Figure 7C). Down-regulation of LOX2 suggests disrupted chloroplast signaling in *pgr5*, which is in line with our previous detection of decreased oxylipin signaling in PSI-photoinhibited *pgr5* (Gollan et al., 2017). Indeed, LOX gene expression is induced by oxylipins (Porta et al., 2008; Sarde et al., 2018), while lipid peroxidation is an early step in enzymatic oxylipin synthesis (Wasternack and Hause, 2013), making it difficult to distinguish the cause of low lipid oxidation from the effect in this case. Equivalent luminescence signals were detected in both WT and *pgr5* after physical wounding of leaves (Figure 7B), indicating that wound-responsive lipid oxidation pathways were operational in *pgr5* plants.

## Conclusion

This study shows that PSI photoinhibition is rapidly induced under conditions of imbalanced reduction pressure between PSI donor and acceptor sides, and that PSI photoinhibition is not associated with increases in HL-induced ROS accumulation at the whole leaf level. It should be noted that the donor/acceptor side imbalance in *pgr5* under HL is more severe than that induced by natural conditions like low temperatures or fluctuating light (Terashima et al., 1994; Kono et al., 2014; Lima-Melo et al., 2019). Therefore the extent, and probably the mechanism, of PSI photoinhibition in the *pgr5* system can be considered overly severe. Nonetheless, the current results can improve our understanding of PSI inhibition induced by natural stresses, while also underscoring the importance of PGR5-dependent protection of PSI. We present the notion that PSI inactivation prevents ROS over-production and oxidative stress in the chloroplast stroma and in the wider cell. This resembles the protective effect of PSII photodamage that prevents over-reduction of downstream components (Tikkanen et al., 2014; Huang et al., 2016), except that damaged PSII is replenished far more efficiently than damaged PSI (Aro et al., 1993; Scheller and Haldrup, 2005). In light of its slow recovery, PSI protection is often considered to be the main target of photosynthetic regulation mechanisms (Tikkanen et al., 2012; Larosa et al., 2018); however, the current work suggests that PSI is also expendable in the effort to mitigate stromal over-reduction. Considering our recent findings that a partially inhibited PSI pool can support normal CO_2_ metabolism (Lima-Melo et al., 2019), inactivation of PSI may be more affordable than commonly thought.

## Data Availability

The raw data supporting the conclusions of this manuscript will be made available by the authors, without undue reservation, to any qualified researcher.

## Author Contributions

YL-M, E-MA, and PG devised the work. YL-M, VA, AL, RS, and PG conducted the experiments. YL-M, MT, E-MA, JS, and PG analyzed the data. YL-M, E-MA, JS, and PG wrote the manuscript.

## Conflict of Interest Statement

The authors declare that the research was conducted in the absence of any commercial or financial relationships that could be construed as a potential conflict of interest.
